# Snake and Spider Toxins Induce a Rapid Recovery of Function of Botulinum Neurotoxin Paralysed Neuromuscular Junction

**DOI:** 10.3390/toxins7124887

**Published:** 2015-12-08

**Authors:** Elisa Duregotti, Giulia Zanetti, Michele Scorzeto, Aram Megighian, Cesare Montecucco, Marco Pirazzini, Michela Rigoni

**Affiliations:** 1 Department of Biomedical Sciences, University of Padua, Via U. Bassi 58/B, 35131 Padova, Italy; elisa.duregotti@gmail.com (E.D.); gzanetti89@gmail.com (G.Z.); scorzeto.michele@gmail.com (M.S.); aram.megighian@unipd.it (A.M.); cesare.montecucco@gmail.com (C.M.); 2 Institute for Neuroscience, National Research Council, Via U. Bassi 58/B, 35131 Padova, Italy

**Keywords:** botulinum neurotoxins, animal neurotoxins, nerve terminals degeneration, mouse, DAS assay, paralysis, neuroexocytosis

## Abstract

Botulinum neurotoxins (BoNTs) and some animal neurotoxins (β-Bungarotoxin, β-Btx, from elapid snakes and α-Latrotoxin, α-Ltx, from black widow spiders) are pre-synaptic neurotoxins that paralyse motor axon terminals with similar clinical outcomes in patients. However, their mechanism of action is different, leading to a largely-different duration of neuromuscular junction (NMJ) blockade. BoNTs induce a long-lasting paralysis without nerve terminal degeneration acting via proteolytic cleavage of SNARE proteins, whereas animal neurotoxins cause an acute and complete degeneration of motor axon terminals, followed by a rapid recovery. In this study, the injection of animal neurotoxins in mice muscles previously paralyzed by BoNT/A or /B accelerates the recovery of neurotransmission, as assessed by electrophysiology and morphological analysis. This result provides a proof of principle that, by causing the complete degeneration, reabsorption, and regeneration of a paralysed nerve terminal, one could favour the recovery of function of a biochemically- or genetically-altered motor axon terminal. These observations might be relevant to dying-back neuropathies, where pathological changes first occur at the neuromuscular junction and then progress proximally toward the cell body.

## 1. Introduction

Botulinum neurotoxins (BoNTs) produced by Clostridia are responsible for the flaccid paralysis of botulism [1,2]. Many different BoNTs are known and are grouped into seven serotypes (BoNT/A to BoNT/G). They include a metalloprotease domain that specifically cleaves three essential components of the synaptic vesicle fusion machinery leading to a persistent, but reversible, blockade of neurotransmission with no morphological alterations of the neuromuscular junction (NMJ) [2,3,4]. Indeed, most botulism patients survive if their respiration is mechanically supported. The duration of BoNTs-induced neuroparalysis depends on the BoNT serotype and on the toxin dose [5]. BoNT/A and BoNT/C induce the longest paralysis (up to many months), whilst BoNT/E and /F cause the shortest one (few weeks) [6,7,8]. The same occurs in rats and mice, although functional recovery is 3–4 times faster than in humans [5,9,10].

A similar peripheral neuroparalysis is also caused by some animal neurotoxins which induce a reversible degeneration of motor axon terminals. Their use provides a relevant model for the molecular characterization of the neurorepair process after injury [11]. These animal presynaptic neurotoxins include α-Latrotoxin (α-Ltx), a pore-forming toxin contained in the black widow spider venom (genus Latrodectus) [12,13], and β-Bungarotoxin (β-Btx, from the Taiwan krait *Bungarus multinctus* venom) [14]. β-Btx belongs to a family of snake neurotoxins endowed with phospholipase A2 activity, named SPANs [15,16]. Despite their different biochemical activities, intoxication by these animal neurotoxins results in a calcium overload inside motor axon terminals that, in turn, triggers a massive neuroexocytosis of synaptic vesicles and the progressive degeneration of the nerve endings [17,18]. Very remarkably, such an effect is strictly limited to the unmyelinated end-plate and is characterized by mitochondria failure and cytoskeletal fragmentation [11,19,20]. Nevertheless, the consequent neuromuscular paralysis is completely reversible: in rodents, nerve terminal regeneration and functional re-innervation are fully restored within a few days [21,22], in a process orchestrated by muscle, Schwann cells, and the basal membrane [23,24]. In humans, the peripheral neuroparalysis induced by the envenomation with snake venoms containing SPANs is functionally reversed within 3–6 weeks [25,26].

Despite the fact that these animal neurotoxins cause a complete disappearance of motor axon terminals, whereas BoNTs do not, the functional recovery in the first case is much faster than in the latter [21,22,27,28,29,30].

Based on these premises, we decided to investigate whether the local administration of α-Ltx or β-Btx could rapidly reverse the otherwise long-lasting effect induced by BoNTs in mice, leading to functional recovery from botulism paralysis. Functional and biochemical read-outs clearly indicate that a single injection of animal neurotoxins switches the kinetics of recovery from several weeks to few days, providing a proof of principle that any form of biochemically/genetically dysfunctional motor axon terminal can be restored by inducing a degeneration/regeneration process.

## 2. Results

### 2.1. α -Latrotoxin or β–Bungarotoxin Injection Accelerates the Recovery from BoNTs-Induced Paralysis

As a first approach to evaluate the effect of animal neurotoxins on the functional recovery of the BoNTs-poisoned NMJs, we took advantage of a well-established model for assessing the kinetics of rescue from BoNTs-induced muscular paralysis: the Digit Abduction Score (DAS) assay. This method is widely used to evaluate the severity and duration of local muscle weakening following the intramuscular injection of BoNTs into mouse hind limbs. We used the two BoNT serotypes most frequently associated to human botulism and that are commercially available for human therapy: BoNT/A and BoNT/B [2,31,32].

**Figure 1:**
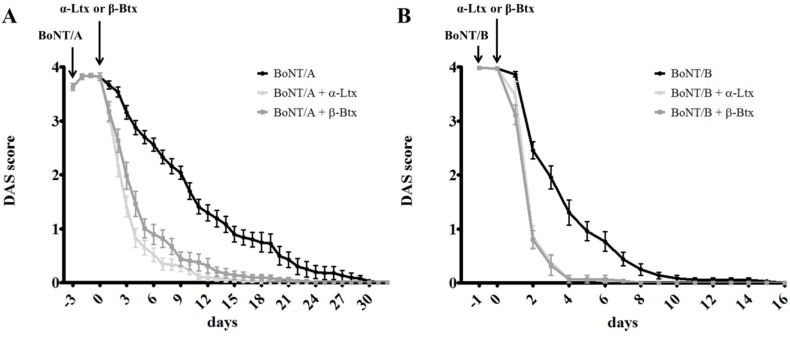
Digit Abduction Score (DAS) assay on single- or double-injected mice. Sub-lethal doses of BoNT/A (**A**) or BoNT/B (**B**) were i.m. injected in mice hind limbs; once complete paralysis was achieved (within 12 h from injection, DAS = 4), two groups of mice received a second i.m. injection of α-Ltx or β-Btx (three days after BoNT/A and 24 h after BoNT/B administration). The rescue from paralysis was monitored over time, until complete recovery was attained (DAS = 0). Representative experiments, *N* = 10 mice for each condition. Error bars represent s.e.m.

As shown by the black trace in Figure 1A, a minimal amount of BoNT/A induces a long lasting paralysis of mice hind limbs. Notably, the effect has a maximum severity (DAS ≥ 3.5) for at least five days, with the subsequent recovery—characterized by a slow, though progressive, increase in the capability of toes to abduct—taking more than 20 days to be substantially completed (DAS ≤ 0.5). The other two traces show that α-Ltx (light gray) and β-Btx (dark gray), injected when BoNT/A has reached its maximum effect (three days after injection, indicated as day zero), significantly shorten the time needed to rescue from paralysis: recovery (DAS ≤ 0.5) is indeed achieved well within nine days. Furthermore, the severity of paralysis drops very quickly from the maximum score, at the time of animal toxin injection, to a very low value (DAS ≤ 1), where hind-limb muscles are still weak but no longer paralyzed, allowing an almost normal control of toe movements. Figure 1B shows that a very similar outcome was obtained using BoNT/B: in this case, even though the maximum severity is likewise achieved, the paralysis lasts much shorter, being substantially extinguished (DAS ≤ 0.5) within seven days. Nevertheless, DAS scores of double-injected mice (α-Ltx or β-Btx 24 h after BoNT/B, indicated as day zero) return to baseline in three days, showing again a significantly faster recovery. The difference between single- and double-injected animals is less remarkable in the case of BoNT/B treated animals with respect to those treated with BoNT/A, but this is the immediate consequence of the different duration of action of the two BoNT serotypes. In fact, BoNT/B-induced paralysis is about three times shorter than that caused by BoNT/A [32].

### 2.2. Synaptic Activity of BoNT-Paralyzed Muscles is Restored Earlier Following α-Latrotoxin or β-Bungarotoxin Injection

Though very reliable, DAS assay provides a more qualitative than quantitative read-out of muscle paralysis. Therefore, to monitor the functional recovery of BoNTs-poisoned nerve terminals in a more quantitative way, we performed electrophysiological recordings (ER) on soleus NMJs of single- or double-poisoned mice at different time points after treatments. The experiment was conducted as in the case of DAS assay, but at indicated times soleus muscles were collected and evoked junction potentials (EJPs) were recorded *ex vivo* in order to determine the functional state of single NMJs. 

**Figure 2:**
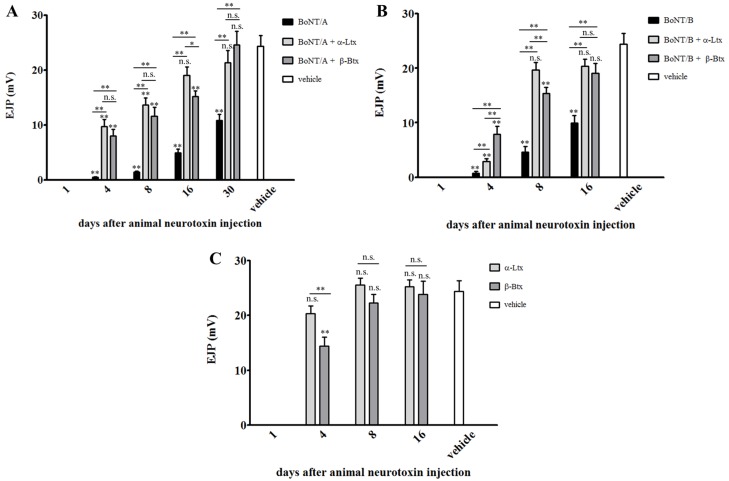
Electrophysiological recordings on single- or double-injected soleus muscles. Sub-lethal doses of α-Ltx or β-Btx were administered i.m. in mice hind limbs previously injected at the same site with BoNT/A (**A**, three days earlier) or BoNT/B (**B**, 24 h earlier). Soleus muscles were collected at different time points and processed for electrophysiological recordings. Muscles injected with BoNTs plus animal neurotoxins recover faster than BoNTs-treated ones. The same analysis was performed on solei injected with animal neurotoxins only (**C**). Bars represent the average EJP amplitude of 45 muscle fibers from three different mice per condition; paired *t*-test, * *p* < 0.01, ** *p* < 0.001 *versus* control (vehicle) or other conditions of the same time point; error bars represent s.e.m. n.s.= not significative.

Figure 2 reports that, upon a supramaximal electric stimulation, neuroexocytosis occurs, as assessed by the recorded post synaptic depolarization (white bar). As expected, and in agreement with the DAS assay, BoNT/A inhibits the release of acetylcholine (Ach) soon after administration, indeed poisoned solei do not generate EJPs (day one, black bar). The same effect is observed at day one in double-injected muscles, as well as in those treated with the sole animal neurotoxins (Figure 2C), and is fully consistent with their degenerating effect on peripheral nerve endings [11]. However, four days after the injection of the animal toxins, which is a time window sufficient for degeneration/regeneration of nerve terminals to occur (Figure 2C), double-injected muscles display a partial restoration of the synaptic activity. The same is not observed in muscles injected with only BoNT/A, which at the same time point are still completely paralyzed (Figure 2A). Importantly, such a profile is maintained in the following time points, where double-injected muscles show EJPs of higher amplitudes compared to those of single-injected ones. Notably, 30 days after animal toxin administration, the functionality of double-injected muscles is substantially restored, while solei treated with only BoNT/A respond to nerve stimulation with an average depolarization which is only about 50% with respect to control ones. This faster recovery of NMJs functionality is not restricted to muscles paralyzed by BoNT/A, but can be extended also to BoNT/B-poisoned ones (Figure 2B), even though in this case the overall rescue profile is faster, as BoNT/B-induced paralysis has, in itself, a shorter duration [32].

As a control, ER were also performed on muscles injected with α-Ltx or β-Btx alone: as shown in Figure 2C, the complete recovery from paralysis is achieved more rapidly than in double-injected muscles (Figure 2A,B). This could be due to some residual activity of BoNTs, which might diffuse away from the site of injection to reach the blood circulation, being therefore able to re-affect regenerated nerve terminals, slightly impairing neurotransmission. This is consistent with the fact that BoNTs can be found in the general circulation of botulism patients for many days after the onset of symptoms development [33,34].

### 2.3. Fluorescence Microscopy Analysis of SNAP25 and VAMP1 Turn-Over at Single- or Double-Poisoned NMJs

It is now well documented that BoNT-induced neuroparalysis is a direct consequence of the specific cleavage of different SNARE proteins by the metalloprotease activity of the toxins. Specifically, BoNT/B removes a large cytosolic fragment of VAMP, thus preventing the formation of the SNARE complex, whereas BoNT/A cleaves few residues from the *C*-terminal of SNAP25: the resulting truncated SNAP25 (t-SNAP25) can still form stable SNARE complexes, which are although unable to mediate neuroexocytosis [3,5]. This can be ascribed to the inability of t-SNAP25 to correctly interact with other SNARE complexes, preventing the assembly of the SNARE super-complex necessary to mediate synaptic vesicles fusion [5,35,36].

Being the blockade of neurotransmission by BoNTs the result of SNARE components proteolysis, we followed the kinetics of SNARE proteins cleavage by means of immunohistochemistry, using appropriate antibodies in order to characterize at a molecular level the effects of animal neurotoxins on BoNTs-poisoned NMJs. Importantly, this analysis was performed on muscles previously processed for ER, permitting a direct comparison of their functional state with SNARE proteins cleavage. For this purpose, in the case of BoNT/A-poisoned muscles we took advantage of a well characterized antibody that only recognizes the BoNT/A-cleaved form of SNAP25 [31], whereas BoNT/B-poisoned ones were labelled with an antibody which only binds the intact form of VAMP1 [37], as this is the main VAMP isoform present at NMJ [38]. As a control of their integrity state, presynaptic nerve terminals were also stained for neurofilaments (NF) and SNAP25 (using an antibody which recognizes both intact and truncated SNAP25, indicated as SNAP25_total_), whereas fluorescent α-Bungarotoxin (α-BTX) was used to visualize post-synaptic specializations.

**Figure 3:**
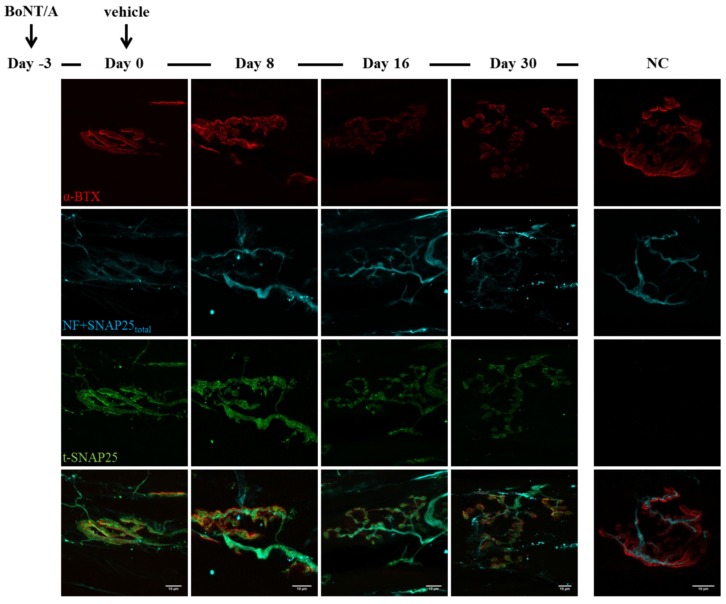
Time course of SNAP25 cleavage by BoNT/A. Soleus muscles injected with BoNT/A were dissected at different time points, analysed by electrophysiology and then processed for indirect immunohistochemistry. Day zero refers to NMJs treated for three days with BoNT/A (at day zero a second injection with animal neurotoxins was performed, see Figure 4 and Figure 5). A strong staining of BoNT/A-cleaved SNAP25 (t-SNAP25) is detectable at NMJs from the very beginning of the analysis, and persists, though with decreasing intensity, until day 30. In untreated muscles, t-SNAP25 is undetectable (NC: negative control, NF: neurofilaments). Bar = 10 µm.

**Figure 4:**
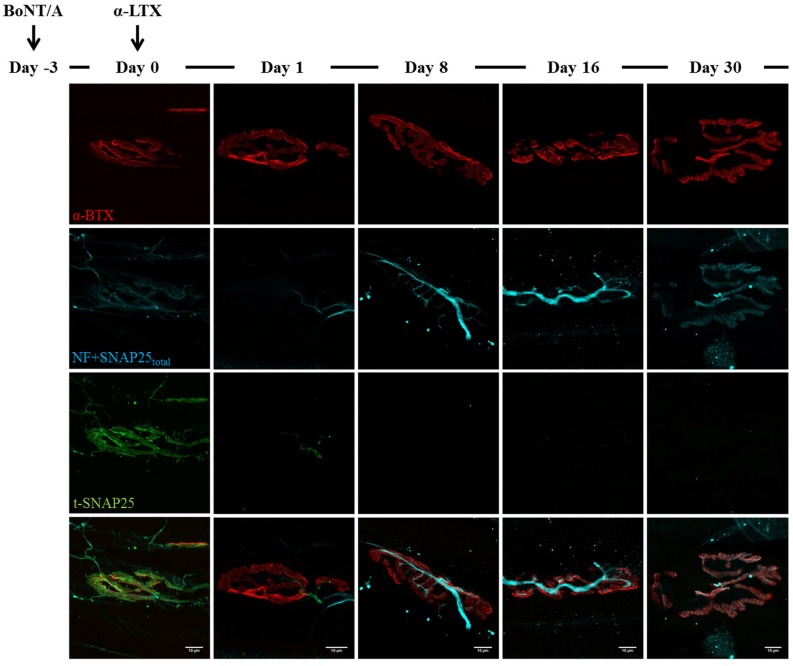
BoNT/A-cleaved SNAP25 turn-over at α-Ltx-injected NMJ. α-Ltx was administered i.m. in mice hind limbs 3 days after the injection of BoNT/A at the same site (day zero). Immunohistochemistry was then performed at different time points on soleus muscles previously processes for electrophysiology. As shown in the panel, the acute degeneration of nerve terminals is induced within 24 h from α-Ltx injection; at day eight, regeneration is achieved as demonstrated by the re-appearance of the SNAP25_total_ and neurofilaments (NF) staining. However, no t-SNAP25 is detectable at regenerated NMJs throughout the time-course of the experiment. Bar = 10 µm.

**Figure 5:**
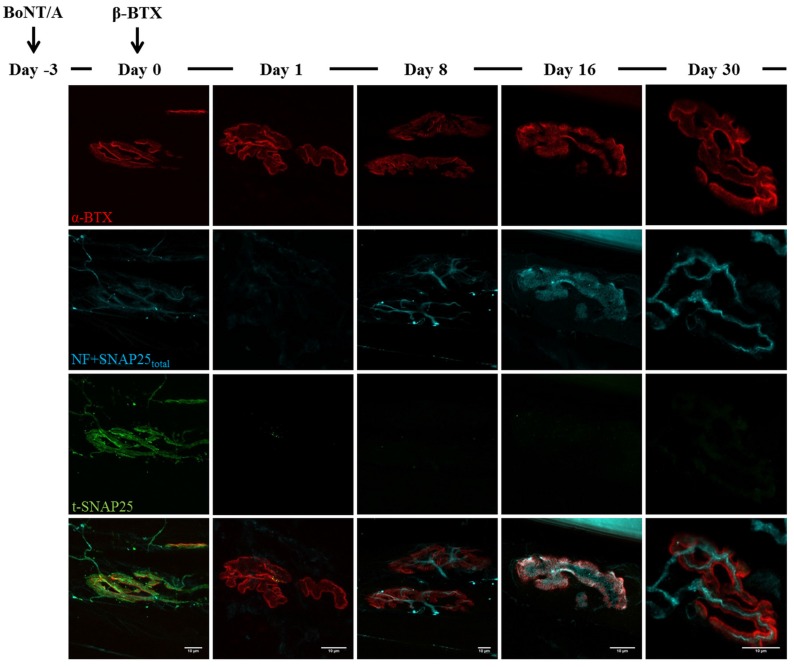
BoNT/A-cleaved SNAP25 turn-over at β-Btx-injected NMJ. β-Btx was administered i.m. in mice hind limbs three days after a first injection of BoNT/A at the same site. Immunohistochemistry was then performed at different time points on soleus muscles previously processes for electrophysiology. Similarly to α-Ltx, β-Btx induces an acute degeneration of nerve terminals within 24 h, followed by a complete regeneration. Again, the staining of t-SNAP25 in no more detectable at regenerated motor axon terminals. Bar = 10 µm.

As shown in Figure 3, soon after BoNT/A injection t-SNAP25 starts accumulating at poisoned NMJs, and the staining persists until the end of the kinetics, although slowly decreasing its intensity. As expected, t-SNAP25 antibody does not cross-react with intact SNAP25, since no staining is detectable at untreated NMJs. As previously reported, chemically denervated NMJs sprout terminal and nodal processes (see Figure 3, day zero), that become longer and widespread over time [39]; in addition, paralyzed NMJs lose their typical and well-defined shape, and the post-synaptic staining of Ach receptors becomes progressively weaker and fragmented. A different scenario arises in double-injected muscles (Figure 4 and Figure 5): 24 h after animal neurotoxins administration (injected three days after BoNT/A), NMJs have degenerated, as proven by the disappearance of neurofilaments, intact SNAP25 and t-SNAP25. Importantly, by day eight all motor axon terminals have regenerated, as shown by the staining of newly-synthetized SNAP25 and NF. Noteworthy, here t-SNAP25 is completely absent, suggesting that the degeneration “cleared” nerve terminals from BoNT/A L chains, neutralizing its poisonous effects. Moreover, the post-synaptic staining of NMJs is much more preserved than in muscles injected with only BoNT/A: this might be due to the trophic effect of Ach, whose release is earlier restored in double-injected solei (Figure 2), thus preventing the disassembly of Ach receptors-clusters forming post-synaptic specializations.

**Figure 6:**
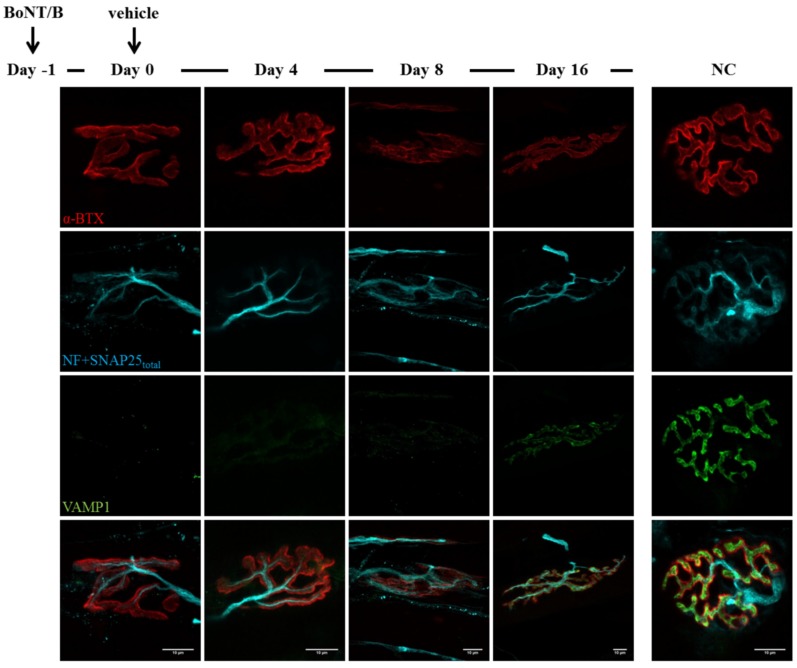
Time course of VAMP1 cleavage by BoNT/B. Soleus muscles injected with BoNT/B were dissected at different time points, analysed by electrophysiology and then processed for indirect immunohistochemistry. Day zero refers to NMJs treated for 24 h with BoNT/B (at day zesro a second injection with animal neurotoxins was performed, see Figure 7 and Figure 8). VAMP1 staining, which is brightly present at untreated NMJs, disappears soon after BoNT/B injection and starts reappearing by day 16. Bar = 10 µm.

**Figure 7:**
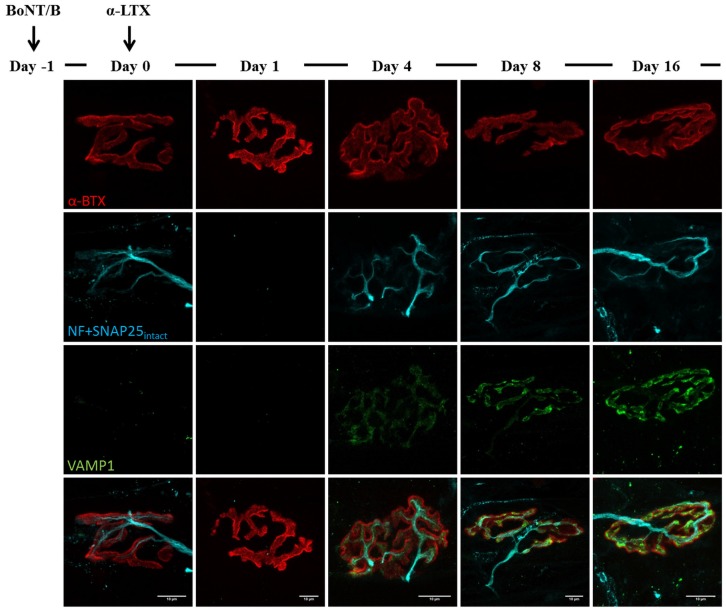
VAMP1 reappearance following α-Ltx injection at BoNT/B-poisoned NMJs. α-Ltx was administered i.m. in mice hind limbs 24 h after BoNT/B injection. Soleus muscles were then processed for immunohistochemistry after electrophysiology. Within 24 h from α-Ltx injection, nerve terminals completely degenerate, as demonstrated by the disappearance of SNAP25_total_ and neurofilaments (NF) stainings; however, by day four newly-regenerated axon terminals show a clear labelling of VAMP1, which becomes more brilliant and defined over time. Bar = 10 µm.

**Figure 8:**
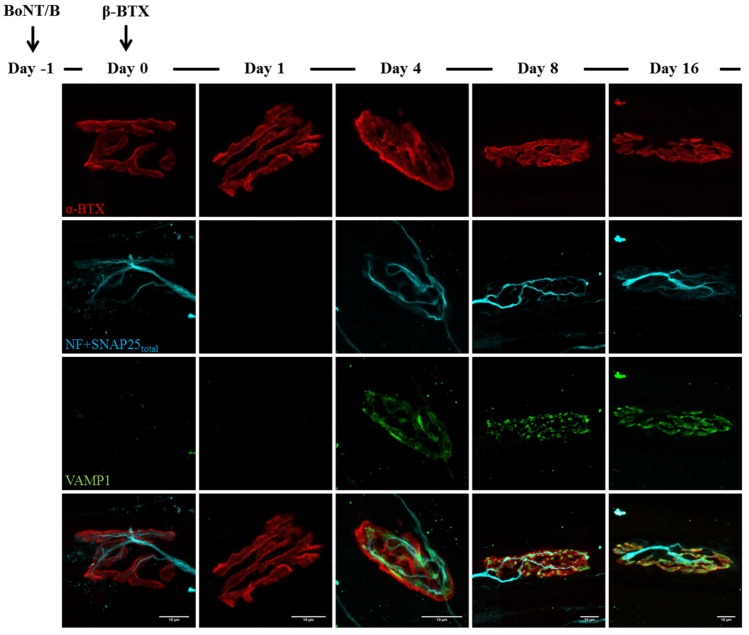
VAMP1 reappearance following β-Btx injection at BoNT/B-poisoned NMJs. β-Btx was administered i.m. in mice hind limbs 24 h after BoNT/B injection. Soleus muscles were then processed for immunohistochemistry after electrophysiological recordings. Similarly to α-Ltx, regeneration of nerve terminals takes place by day four and is paralleled by the re-appearance of VAMP1 staining at NMJs. Bar = 10 µm.

Similar outcomes are observed in BoNT/B-treated mice: as expected, VAMP1 staining disappears soon after BoNT/B injection, and newly synthetized VAMP1 starts to be detectable starting from day 16 (Figure 6). Again, many neuronal sprouts can be seen at late time points, when paralyzed NMJs also become elongated and shapeless, though to a lesser extent than those treated with BoNT/A. When α-Ltx or β-Btx are administered, nerve terminals degenerate, and only the post-synaptic labelling is detectable at NMJs (Figure 7 and Figure 8, day one). However, a rapid and complete regeneration takes place by day four, as assessed by the reappearance of the presynaptic markers SNAP25 and neurofilaments, as well as of VAMP1. Notably, the synaptic activity recovery parallels the reappearance of VAMP1 staining, which becomes more and more brilliant over time reaching control level by day 16, when NMJs perform indistinguishably from that of control muscles (Figure 2B).

## 3. Discussion

Several peripheral human pathologies are due to biochemical lesions of motor axon terminals, caused by a genetic alteration or by exogenous agents. In non-cell autonomous and dying-back axonopathies such as ALS (amyotrophic lateral sclerosis) and autoimmune neuropathies, many molecular changes influencing motor neurons degeneration occur at the NMJ at the very early stages of the disease, and then progress along the axon leading to denervation and irreversible paralysis [40,41,42,43]. Here, we aimed at providing a proof of principle that a NMJ, biochemically-lesioned in its motor axon terminal, can be returned to functionality by operating a surgical removal of the terminal itself, followed by its re-growth under the stimulus and guidance of the basal membrane, the perisynaptic Schwann cells and the muscle fibre. As a biochemical damage, we chose one which has been well defined in the last 20 years, *i.e.*, the cleavage of a SNARE protein by a BoNT, which leads to long lasting but totally reversible paralysis of peripheral nerve terminals [2]. We have extended the study to the two BoNT serotypes that are the main responsible of human botulism and, at the same time, are used in the therapy of human pathological conditions characterized by hyperfunctionality of peripheral nerve terminals [32,44,45,46]. BoNT/A cleaves the C-terminus of SNAP25, whilst BoNT/B removes the major part of the cytosolic domain of the integral synaptic vesicle protein VAMP [3,16]. Both proteins are essential components of the SNARE complex, the nanomachine which mediates neurotransmitter release: for this reason, their cleavage leads to the persistent paralysis that is the hallmark of botulism [5,47,48]. To perform the surgical removal of the motor axon terminals, we used two neurotoxins that bind specifically to the presynaptic plasma membrane, altering its permeability and allowing rapid influx of calcium to the cytosol; this in turn triggers a series of events, only partially known, leading to a complete degeneration which is spatially-restricted to nerve terminals only, with no evident damage of the axon [11,13,15,22,49].

Double poisoning with black widow spider venom and partially-purified preparations of BoNT/A was performed before in experiments aimed at understanding their mechanism of action, which was only partially known at that time [50,51]. In two other studies, the reversibility of BoNT/A induced paralysis was studied by inducing NMJ regeneration upon crushing the nerve terminal [52,53]. A similar approach was also extended to some *in vitro* experiments, in which α-Ltx was found to restore SNAP25 by elimination of the SNAP25 cleaved by BoNT/A. Nevertheless, this study did not include *in vivo* observations, therefore not providing any information about the effect of the spider toxin on the functional recovery of BoNTs-paralyzed NMJs [54].

The present work takes advantage of the actual wider knowledge of the mechanism of action of all the neurotoxins used here [2,11,12,15,16], including PLA2 neurotoxins (SPANs) and BoNT/B, which have never been tested in such double poisoning experiments before. 

The remarkable finding described here is that, notwithstanding the target cleaved by the BoNTs, both α-Ltx and β-Btx are capable of effectively shorten the duration of the paralysis caused by BoNTs in mice, at doses that exclude a systemic effect. The progressive disappearance of the peripheral paralysis is accompanied by a recovery of the NMJ function, as assessed by electrophysiological measurements. In addition, we documented by fluorescence microscopy with specific antibodies that BoNT/A paralysis and recovery are paralleled by appearance and disappearance of the truncated form of SNAP25; a similar relation was found between VAMP1 staining and blockade of the motor axon terminals in BoNT/B treated animals. Moreover, to the best of our knowledge, this is the first time that the direct effect of BoNT/B on its substrate VAMP1 has been immunohistochemically characterized in terms of onset and resolution.

The general and relevant conclusion that can be drawn by the present study is that biochemical lesions of motor axon terminals, associated with a loss of synaptic functionality, can be overcome by treatments that cause a reversible degeneration of the terminals themselves. As the NMJ is one of the few anatomical structures whose regeneration capacity has been retained through evolution [23,24], the degeneration and removal of the motor axon terminal is followed by its regeneration and repositioning on the basal membrane, with complete regain of function. In the light of these observations, it would be interesting and relevant to extend the present approach to diseases characterized by a chronic and severe dysfunction of the motor axon terminals, such as ALS and some autoimmune neuropathies. 

## 4. Experimental Section

### 4.1. Animals and Toxins

Experiments were performed on Swiss-Webster adult male CD1 mice (Plaisant Srl) in accordance with the Council Directive 2010/63/EU of the European Parliament, the Council of 22 September 2010 on the protection of animals used for scientific purposes, and approved by the Italian Ministry of Health (authorization number 359/2015, 11 May 2015).

BoNT/A was prepared and purified as previously described [55,56], BoNT/B was produced in *E. coli* via recombinant methods [57]. α-Ltx and β-Btx were purchased from Alomone (Jerusalem, Israel) and Sigma-Aldrich (St.Louis, MO, USA), respectively. Their purity was checked by SDS page and their potency tested in *ex vivo* hemidiaphragm preparations [15]. 

BoNT/A and BoNT/B were diluted with physiological saline (0.9% NaCl plus 0.2% gelatine) to a final concentration of 0.25 pg/µL and 0.5 pg/µL respectively, and locally injected in the left mouse hind-limb (1 µL/g of weight), in order to reach the final intramuscular dose of 0.25 ng/kg (BoNT/A) or 0.5 ng/kg (BoNT/B). Similarly, at indicated time, α-Ltx (5 μg/kg) or β-Btx (10 μg/kg) were injected at the same site. Control animals were injected with saline. All injections were performed upon isofluorane anaesthetization of two months-old mice weighting around 20–25 g. Treated mice underwent DAS score evaluation and electrophysiological recordings at defined time points, as described in the following sections.

### 4.2. Digit Abduction Score Assay (DAS)

Swiss–Webster adult male CD1 mice were housed under controlled light/dark conditions, and food and water were provided *ad libitum*. The degree of hind limb paralysis was evaluated by the Digit Abduction Score Assay (DAS), which measures the local muscle weakening following BoNTs injection into mouse hind-limb [58,59]. Briefly, the local paralysis was scored on a five point scale, with 0 corresponding to a normal abduction of all digits of the hind limbs and four corresponding to the maximum degree of paralysis, *i.e.*, none of the toes can abduct. Ten mice for each condition were employed. Treated mice were checked once per day until the complete recovery of abduction capability.

### 4.3. Electrophysiological Recordings (ER)

Treated mice were sacrificed at scheduled times by anesthetic overdose followed by cervical dislocation, soleus muscles were dissected, subjected to electrophysiological measurements and then fixed for immunohistochemistry. Three mice were used for each condition at each time-point. Electrophysiological recordings (ER) were performed in oxygenated Krebs-Ringer solution on sham or neurotoxins-injected soleus muscles, using intracellular glass microelectrodes (WPI) filled with one part 3 M KCl and two parts 3 M CH3COOK. Evoked junction potentials (EJP) were recorded in current-clamp mode, starting from resting membrane potential of −70 mV, adjusted with direct current injection when needed. EJPs were elicited by supramaximal nerve stimulation at 0.5 Hz, using a suction microelectrode connected to a S88 stimulator (Grass, Warwick, RI, USA). To prevent muscle contraction after dissection, samples were incubated for 10 min with 1 μM μ-Conotoxin GIIIB (Alomone, Jerusalem, Israel). Signals were amplified with intracellular bridge mode amplifier (BA-01X; NPI, Tamm, Germany), sampled using a digital interface (NI PCI-6221; National Instruments, Austin, TX, USA) and recorded by means of electrophysiological software (WinEDR; Strathclyde University, Glasgow, Scotland, UK). EJPs measurements were carried out with Clampfit software (Molecular Devices, Sunnyvale, CA, USA).

### 4.4. NMJ Immunohistochemistry (IHC)

At the end of ER, soleus muscles were immediately fixed in 4% (*wt/vol*) PFA in PBS for 30 min at RT. Samples were quenched in 50 mM NH4Cl in PBS, then permeabilized and saturated for 2 h in blocking solution (15% *vol/vol* goat serum, 2% *wt/vol* BSA, 0.25% *wt/vol* gelatin, 0.2% *wt/vol* glycine in PBS), containing 0.5% Triton X-100. Incubation with the following primary antibodies was carried out for at least 48 h in blocking solution: anti-SNAP25 (SMI81 mouse monoclonal, 1:100, BioLegend, San Diego, CA, USA), anti-neurofilaments (mouse monoclonal, anti-NF200, 1:200, Sigma-Aldrich, St. Loius, MO, USA), anti-VAMP1 (rabbit polyclonal 1:200, generated as described in [37], and anti-SNAP25 BoNT/A-cleaved (t-SNAP25, rabbit polyclonal 1:200, generated as described in [31]. Muscles were then extensively washed and incubated with the appropriate secondary antibodies (Alexa-conjugated, 1:200 in PBS, Thermo Scientific, Waltham, MA, USA) supplemented with Alexa555-conjugated α-Btx (1:200, Thermo Scientific, Waltham, MA, USA) to counterstain post-synaptic nicotinic acetylcholine (Ach) receptors. Images were collected with a Leica SP5 confocal microscope (Leica Microsystems, Wetzlar, Germany) equipped with 100X HCX PL APO NA 1.4 objective. Laser excitation line, power intensity, and emission range were chosen according to each fluorophore in different samples to minimize bleed-through. 
